# Nicotine pre-exposure reduces stroke-induced glucose transporter-1 activity at the blood–brain barrier in mice

**DOI:** 10.1186/s12987-015-0005-y

**Published:** 2015-04-29

**Authors:** Kaushik K Shah, Purushotham Reddy Boreddy, Thomas J Abbruscato

**Affiliations:** Texas Tech University Health Sciences Center, 1300S Coulter, School of Pharmacy, Department of Pharmaceutical Sciences, Amarillo, TX 79106 USA; National Center for Cell Science (NCCS), Cancer Biology, Laboratory No. 6, Pune, 411007 Maharashtra India

**Keywords:** Stroke, Ischemia/reperfusion, Glucose transporter-1, Nicotine, Tobacco smoke, Focal ischemia, Middle cerebral artery occlusion, E-cigarettes

## Abstract

**Background:**

With growing electronic cigarette usage in both the smoking and nonsmoking population, rigorous studies are needed to investigate the effects of nicotine on biological systems to determine long-term health consequences. We have previously shown that nicotine exerts specific neurovascular effects that influence blood brain barrier (BBB) function in response to stroke. In this study, we investigated the effects of nicotine on carrier-mediated glucose transport into ischemic brain. Specifically, the present study investigates glucose transporter-1 (GLUT1) function and expression at the BBB in a focal brain ischemia model of mice pre-exposed to nicotine.

**Methods:**

Nicotine was administrated subcutaneously by osmotic pump at the dose of 4.5 mg/kg/day for 1, 7, or 14 days to reflect the plasma levels seen in smokers. Ischemic-reperfusion (IR) injury was induced by 1 h transient middle cerebral artery occlusion (tMCAO) and 24 h reperfusion. Glucose transport was estimated using an *in situ* brain perfusion technique with radiolabeled glucose and brain vascular GLUT1 expression was detected with immunofluorescence.

**Results:**

The nicotine pre-exposure (1, 7 & 14 day) resulted in significant reduction in D-glucose influx rate (*K*_*in*_) across the BBB, with a 49% reduction in 14 day nicotine-infused animals. We observed a 41% increase in carrier-mediated glucose transport across the BBB in saline-infused tMCAO animals compared to saline-infused sham animals. Interestingly, in the tMCAO group of animals pre-exposed to nicotine for 14 days had significantly attenuated increased glucose transport by 80% and 38% compared to saline-infused tMCAO and sham animals respectively. Furthermore, immunofluorescence studies of GLUT1 protein expression in the brain microvascular endothelium confirmed that GLUT1 was also induced in saline-infused tMCAO animals and this protein expression induction was reduced significantly (*P* < 0.01) with 14 day nicotine pre-exposure in tMCAO animals.

**Conclusions:**

Nicotine pre-exposure reduced the IR-enhanced GLUT1 transporter function and expression at the BBB in a focal brain ischemia mouse model. These studies suggest that nicotine exposure prior to stroke could create an enhanced glucose deprived state at the neurovascular unit (NVU) and could provide an additional vulnerability to enhanced stroke injury.

## Background

In recent years, cigarette smoking has been considered the second-leading risk factor for death and, in the United States, it increases the risk of stroke by 2–4 fold [1]. In addition to cigarette smoking, electronic nicotine vaping device or “e-cigarette” usage has increased in recent years. A recent study in Addiction reports that e-cigarette usage is growing in both populations of former smokers or current smokers as an aid to cut down or quit smoking [2]. Even though it is known that e-cigarettes may provide a healthier option compared to tobacco cigarettes with respect to carcinogens, the effects of long-term exposure to sometimes variable doses of nicotine from e-cigarettes is yet to be determined and will need to be validated with longitudinal studies. Additionally, stroke has become a fourth leading cause of death and disability in the US [1]. Occlusion of a major cerebral artery by an embolus or thrombosis can result in transient or permanent deprivation of nutrient and oxygen supply to parts of the brain. The brain relies heavily on a continuous glucose supply that is regulated across the blood–brain barrier (BBB) via glucose transporters to provide the fuel to maintain cellular ATP as an energy source for brain activity [3-5]. Subsequent deficiency in the major obligatory brain fuels, glucose and oxygen, elicits a number of important neurochemical mechanisms (e.g. excitotoxicity, oxidative stress and inflammation) which can lead to irreversible brain damage. This changing brain microenvironment is tightly regulated by the brain microvasculature which functions to segregate the blood from brain interstitial fluid. The endothelial cells of the BBB provide a dynamic interface between the blood and central nervous system (CNS), maintaining brain homeostasis by selectively limiting the passage of solutes/nutrient/ions from the circulating blood into and out of the brain and it plays an important role in determining the fate of brain tissue after stroke. Brain microvascular endothelial cells working in concert with astrocytes, pericytes and neurons form a neurovascular unit (NVU). Many of these key solutes and nutrients enter the brain by transcellular diffusion and others through passive or active carriers that may utilize receptor-mediated endocytosis [3].

In stroke, loss of blood supply increases energy demand causing the nutrient and ion transporter activity to adapt to deprived conditions. Any changes in the BBB dynamic function resulting from altered function/expression of solute/ion transporters can worsen brain pathophysiology in a number of neurological diseases and disorders including stroke [6,3]. Cerebral glucose transport and metabolic derangement during ischemia have been observed in both animals [7] and human studies [8]. An initial increase in glucose metabolism/utilization occurs due to release of excitatory amino acids in response to ischemic insults, followed by significant reduction in glucose metabolism in the same brain regions with consequent increase in function and expression of glucose transporters at the BBB and in brain [9,10]. Enhanced glucose transporter levels have been suggested to compensate for the lack of glucose availability to the brain in ischemic conditions. Several mechanisms have been suggested to substantiate the adaptive increases in the glucose transporter expression during IR injury. It has been shown that the regulation of GLUT1expression in ischemic brain endothelial cells can occur through a activation of phosphoinositide-3 kinase (PI3K)/Akt pathway via vascular endothelial growth factor [11], HIF1α activation [12] etc.

Changes in BBB function due to nicotine and the components of tobacco smoke can also have significant effects on brain injury [13-16]. The observed effects of chronic nicotine exposure on brain and BBB function are seemingly mostly detrimental during stroke. Nicotine has been reported to cause changes in BBB function that include alterations in expression or function of BBB-associated proteins [17,14], cerebrovascular blood flow [18], BBB permeability [19,13], increases in cerebrovascular thrombosis [20] and an increased post-ischemic inflammatory response [15]. Interestingly, both acute and chronic administration of nicotine has been shown to decrease glucose transport rates across the BBB but increase global glucose utilization in normal rat brain [21-24]. Duelli et al., (1998), have reported decreased 3-O-[14C] methylglucose transfer across the BBB in rats infused with nicotine for 1 week. Those investigators suggested that an increase in local cerebral glucose utilization and reduced glucose transfer rate is associated with an increase in GLUT1 glucose transporter in rats pre-exposed to nicotine for one week [22,23]. Those studies utilized an *in vivo* method to estimate glucose influx and efflux rate constants by injecting the radiotracer IV and analyzing radiotracer amount in blood and brain of animals at set time points. Similar reports in humans are sparse and have demonstrated an overall small reduction in global cerebral glucose utilization although several brain regions showed relative enhanced glucose utilization [25,26]. Moreover, none of the existing studies have investigated the effects of nicotine on glucose transport rate and expression at the BBB during stroke. Thus, to identify an effect of nicotine on BBB glucose transport rate during stroke, we focused our investigations on the validation of these previous findings by utilizing the *in situ* brain perfusion method. This allowed estimation of the initial rate of glucose influx across the BBB under equilibrium conditions with no systemic interference [27]. Further, we also studied the nicotine-induced changes in expression of GLUT1 during stroke to help explain some of the possible nicotine and/or smoking-related changes in cerebrovascular functions in both normal and ischemic brain. Specifically, our studies evaluated BBB glucose transporter function and expression during ischemic stroke in nicotine pre-exposed animals.

## Materials and methods

### Experimental animals

All animal experimental protocols were approved by the Institutional Animal Care and Use Committee of Texas Tech University Health Sciences Center and were conducted in accordance with the National Institute of Health (NIH) *Guide for the Care and Use of Laboratory Animals* (Institute of Laboratory Animal Resources, 1996). A total of 56 CD-1 male mice (Charles River Laboratories, Inc. Wilmington, MA, USA) weighing in the range of 25–35 gm were maintained on 12:12 h light/dark cycle, 23° ± 1°C and used for the experimental purposes.

#### Nicotine treatment

For continuous nicotine infusion (4.5 mg/kg/day), nicotine ((−)-nicotine tartrate, Fisher Bioreagents) was delivered by Alzet osmotic pump (model 2004, Durect Corporation, Cupertino, CA, USA) with an infusion rate of 0.25 μl/h. Pumps were filled with nicotine in saline at a concentration sufficient to deliver 4.5 mg/kg/day over 1, 7 or 14 days. A control group, the sham animals were infused with saline-filled pumps over 14 days. Pumps were surgically implanted under aseptic conditions. In brief, mice were initially anesthetized with 4% isoflurane by inhalation and subsequently maintained under anesthesia with 1.5% isoflurane in N_2_O/O_2_ mixture (70/30) using a SurgiVet Vaporizer (Smiths Medical North America, Waukesha, WI, USA). Mice were placed in the prone position, skin was shaved, disinfected with betadine solution and pumps were inserted subcutaneously by making a small incision between the scapulae and surgically sutured to close the incision. To prevent infection, a topical antibiotic was applied twice daily.

#### *In vivo* MCAO focal brain ischemia model

Fourteen day nicotine- or saline-infused animals were randomly assigned for tMCAO or sham surgery. Transient MCAO was performed on mice as described previously [28,29]. Mice were anesthetized with 4% isoflurane by inhalation and subsequently maintained under surgical anesthesia with 1.5% isoflurane in an N_2_O/O_2_ mixture (70/30) using a SurgiVet Vaporizer. Body temperature was monitored with a rectal temperature probe (RET-3) and maintained 36.5°-37°C throughout surgery using feedback-regulated thermostatic blanket and lamp (TCAT-2DF animal temperature controller, Physitemp Instruments, Inc. Clifton, NJ, USA). In each mouse, local cerebral blood flow (LCBF) was monitored through the skull over the left middle cerebral artery region (MCA; 1 mm posterior and 3 mm lateral to the Bregma) before and 5 min after occlusion of the MCA, and immediately before and ~15 min after reperfusion using a laser Doppler monitor with needle probe (moorLAB, Moor Instruments, Wilmington, DE, USA). Surgery was performed using a Zeiss OPMI Pico I (Carl Zeiss GmbH, Jena, Germany) surgical microscope. In brief, a midline incision on the ventral side of the neck was made after aseptic preparation of the surgical site using a skin disinfectant, betadine. The left carotid bifurcation was accessed and branches of the external carotid artery (ECA), occipital and superior thyroid arteries, were exposed, electrocoagulated (ME102, KLSMartin group, Tuttlingen, Germany) and cut. The left common (CCA) and internal (ICA) carotid arteries were carefully separated from the adjacent tissue and the vagus nerve. The CCA and ICA were clamped using a temporary atraumatic clip, the left ECA was electrocoagulated distally and ligated proximal to the CCA bifurcation using silk suture. A 6-O nylon monofilament suture with a rounded tip (0.18-0.20 mm) was introduced into the CCA by making a small incision just above the ligation on ECA and secured by tightening the silk suture. The ECA was cut distally from the coagulated area and the nylon filament was pulled back and a temporary ICA clip was removed and the filament was inserted into the ICA through the ECA stump and gently moved forward (8.5-9 mm) towards the origin of the MCA followed by removal of the temporary clip from the CCA. A successful MCA occlusion was documented by a sudden fall in local CBF to ≤ 25% of baseline within 5 min of filament insertion and the nylon filament was secured in the place by ligation. After 60 min of MCAO, the mice were reperfused for 24 h by withdrawing the filament and ligating the ECA below the incision. The recovery of local CBF in the MCA regions was observed ~15 min after reperfusion. After successful reperfusion, the incision was sutured and animals placed in a temperature-controlled cage for 2 h and observed for recovery. Sham operated animals underwent the same procedure but without MCA occlusion. After 24 h of reperfusion, a group of tMCAO (n = 5-6) or sham (n = 5-6) animals was used for subsequent *in situ* brain perfusion, and an additional group was prepared for immunohistochemistry (n = 4-5).

#### *In situ* brain perfusion

After 24 h reperfusion, the transport of glucose across the BBB was measured using the *in situ* brain perfusion technique as described previously [27]. Briefly, CD-1 mice were anesthetized by intraperitoneal (*i.p*.) injection of ketamine–xylazine (140–8 mg/kg). The left external carotid artery, superior thyroid arteries and occipital artery were electrocoagulated. The left common carotid was ligated distally and a temporary atraumatic clip was placed proximal to the bifurcation. A small incision was created in the CCA above the ligation and the catheter (PE-10) tubing filled with heparin (25 units/mL) was inserted through the incision. The tube was secured with a suture and the vascular clip removed. The thorax and diaphragm was cut open, the heart ventricles were cut immediately before start of the CCA perfusion. The carotid was initially perfused (2.5 mL/min) for 30 sec with gassed (95% O_2_ and 5% CO_2_) bicarbonate buffer containing 0.5 mM nonradiolabled D-glucose, to create equilibrium condition and immediately with bicarbonate buffer with [^3^H] D-Glucose (0.5 μCi/mL) and vascular permeability markers [^14^C] sucrose (0.1 μCi/mL) for 20 sec. We pre-perfused the brain capillary bed with a known amount of cold D-glucose to estimate glucose kinetics under equilibration state and this minimized possible trans-stimulation transport from varying intracellular glucose concentration [30]. We also used a 20 sec perfusion time to minimize possible back flux and/or metabolism of D-glucose. This method allowed us to measure precisely glucose transport across the BBB in mice during the ischemic insult with and without prior nicotine exposure. Buffer temperature was maintained at 37°C throughout the experiment. At the end of the perfusion time, mice were decapitated and the left brain hemisphere was immediately removed and placed on ice and weighed. Collected brain tissue was digested in 1 mL tissue solubilizer Solvable™ (Perkin Elmer Inc., Waltham, MA, USA) at 55°C overnight, cooled, and mixed with 5 mL scintillation fluid. For each sample, dual label counting was performed simultaneously in a scintillation counter for 10 min. Vascular volume (*V*_*v*_*;* mL.g^−1^), initial transport rate (*K*_*in*_*; mL. sec*^*−1*^*. g*^*−1*^), and flux (*J*_*in*_*; μmol. sec*^*−1*^*. g*^*−1*^) parameters were calculated by the following formula as previously described [31].

1 $$ {K}_{in}=\left\{\frac{\left({Q}_{tot}-\left({V}_v\times {C}_{pf}\right)\right)}{C_{pf}\times T}\right\} $$

2 $$ {J}_{in}={K}_{in}\times {C}_{pf} $$

3 $$ {V}_v = \left(\frac{Q_{tot}}{C_{pf}}\right) $$

Where *Q*_*tot*_ = total amount of solute in brain (mass/weight brain), *C*_*pf*_ = the concentration of solute in the perfusion fluid (g/mL). *V*_*v*_ = brain vascular volume (mL/g), and *T* = perfusion time (sec).

#### Immunohistochemistry

Mice were deeply anesthetized with ketamine-xylazine (140–8 mg/kg; i.p) and were perfused intra-cardially with ice cold PBS for 5 min and flash frozen immediately in chilled isopentane. Sections were cut at 20 μm thick using a cryostat, collected on gold plus slides (Fisher Scientific Company LLC., Hanover Park, IL, USA) and stored at −80°C until processing for immunostaining. Sections were fixed with 4% paraformaldehyde for 15 min at room temperature and washed 3 times each with PBS for 5 min. Subsequently, sections were blocked with 5% donkey serum containing 0.3% triton X-100 in PBS for 1 h at room temperature. Sections were then incubated with rabbit polyclonal anti-GLUT1 antibody (1:300, cat no.SC-7903, Santa Cruz Biotechnology, Inc. Dallas, TX, USA) and vascular marker mouse polyclonal anti-claudin-5 (1:500, cat no. 35–2500, Life Technologies, Grand Island, NY, USA) at 4°C overnight. After incubation, sections were washed 3 times with PBS for 5 min each and incubated with donkey anti-rabbit Alexa 594 (1: 1000) and donkey anti-mouse Alexa 488 (1:1000) for 2 h at room temperature. Sections were washed again 3 times with PBS for 5 min and slides were air dried for 5 min. Sections were then carefully coverslipped with Prolong Gold (anti-fade with DAPI). All specimens were then observed for immunofluorescence using Nikon Eclipse Ti-E Epi-Fluorescence microscope with 20X objective lens. Images were captured and mean total fluorescence intensity was calculated for each color channel using NIS elements AR software and GLUT1 intensity was expressed relative to claudin-5.

#### Statistical method

Data are represented as mean ± SEM and values were statistically analyzed using one way ANOVA analysis of the variance and Bonferonni’s *post hoc* multiple comparison (Prism version 6.05, Graph Pad Software, Inc, San Diego, CA, USA) test. Differences in *P* values less than 0.05 were considered statistically significant*.*

## Results

### Nicotine reduces glucose transport across the BBB

**Figure 1:**
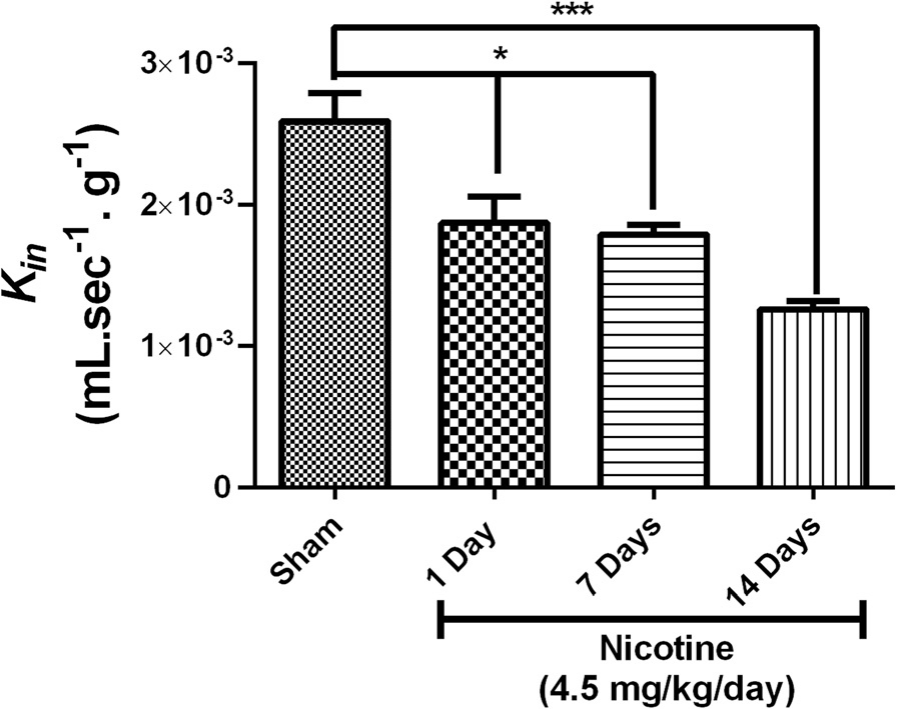
Time-dependent effect of nicotine exposure on glucose transport across the BBB in normal animals. [^3^H] D-Glucose transport (*K*_*in*;_ mL.sec^−1^.g^−1^) into brain was measured using *in-situ* brain perfusion. Animals were pre-exposed to nicotine for 1, 7 and 14 days. Data represent mean ± SEM, n = 5–6 animals, *P < 0.05, and ***P < 0.001 comparing the nicotine-treated groups and the corresponding sham group infused with saline for 14 days, using one way ANOVA analysis of the variance and Bonferonni’s multiple comparison test.

We initially evaluated the glucose transport influx rate at different time intervals after nicotine exposure in normal animals. Test group animals received 4.5 mg/kg/day nicotine for 1, 7, or 14 days and the sham group received saline alone. Glucose transport was estimated on day 1, 7 and 14 of nicotine exposure with [^3^H] D-glucose as a substrate using *in situ* brain perfusion. We observed a BBB influx rate (*K*_*in*_) of 0.0026 ± 0.00069 (mL.sec^−1^.g^−1^) in the saline infused sham group. Animals with nicotine pre-exposure for 1, 7 and 14 days showed significantly reduced D-glucose transport *K*_*in*_ across the BBB compared to the saline-infused sham group (Figure 1).

#### Pre-exposure to nicotine for 14 days reduces ischemic-reperfusion-enhanced glucose transport rate

**Figure 2:**
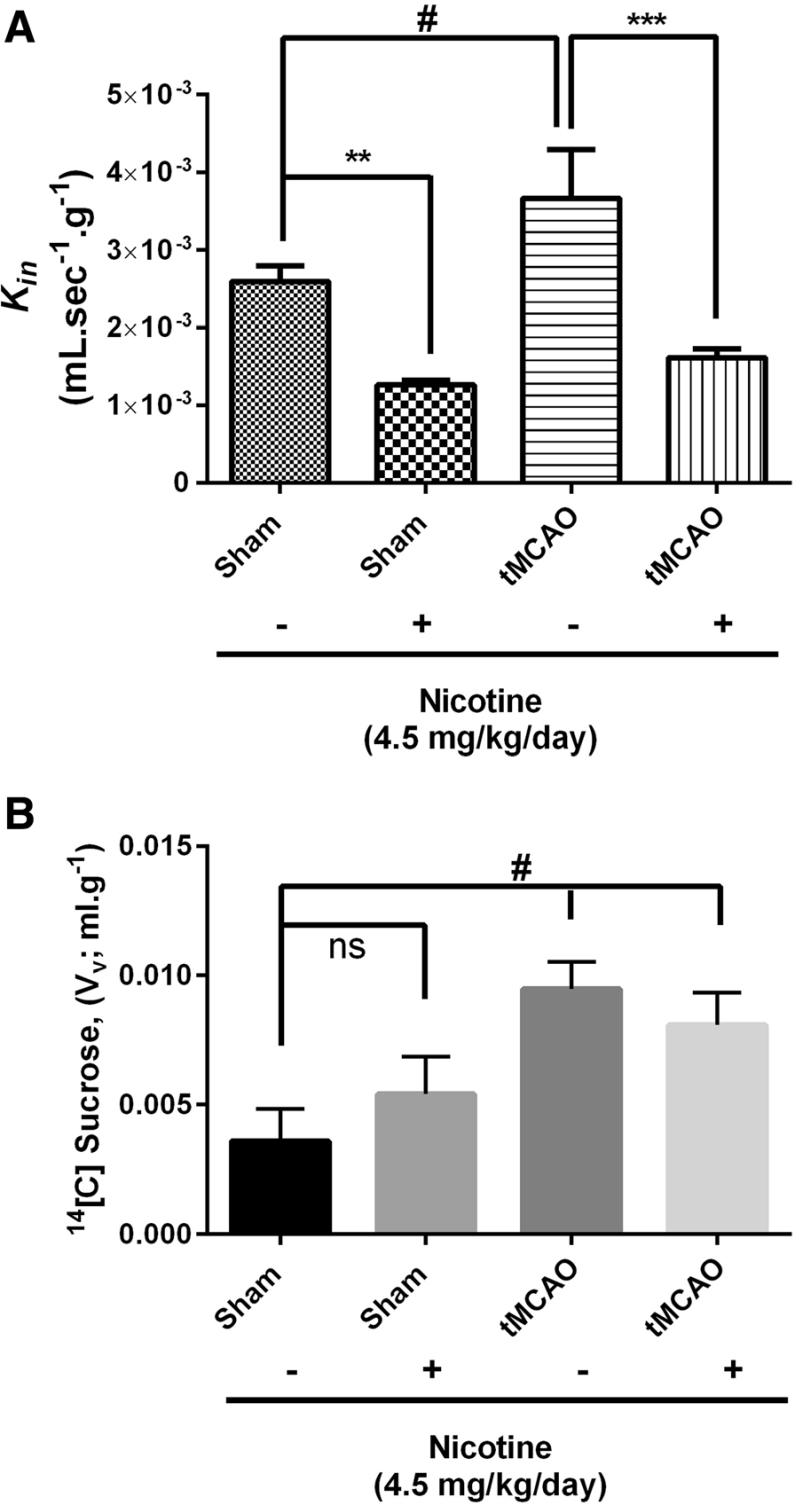
Pre-exposure to nicotine for 14 days reduces ischemic-reperfusion-enhanced glucose transport rate. **(A)** [^3^H] D-Glucose transport (*K*_*in*;_ mL.sec^−1^.g^−1^) in normal and tMCAO animals. **(B)** ^14^[C]-Sucrose vascular volume (*V*_*v;*_ mL. g^−1^). Data represent mean ± SEM, n = 5–6 animals, *P < 0.05 **P < 0.01and ***P < 0.001 comparing the nicotine-infused group with the corresponding saline-infused sham group, ^#^ *P* < 0.05 comparing the nicotine-infused tMCAO group and saline-infused sham group using one way ANOVA analysis of the variance and Bonferonni’s multiple comparison test.

In this study, we investigated the glucose transport influx rate across BBB in tMCAO animals with or without pre-exposure to nicotine and compared these to sham group animals with or without pre-exposure to nicotine. In the saline-infused tMCAO group the glucose transport was significantly increased with *K*_*in*_ of 0.0037 ± 0.00126 (mL.sec^−1^.g^−1^) (*P* < 0.05) compared to saline-infused sham animals. Additionally, we observed a statistically significant (*P* < 0.001) decrease in glucose transport *K*_*in*_ of 0.0016 ± 0.00028 (mL.sec^−1^.g^−1^) across the BBB in nicotine pre-exposed tMCAO animals compared to the saline-infused tMCAO group. These studies suggest a nicotine regulatory effect on glucose transporter function in both normal physiology and ischemic-reperfusion conditions (Figure 2A; *see* Table 1.). Further, the observed changes in glucose transport across BBB were independent of changes in vascular volume or permeability as measured simultaneously in these experiments (Figure 2B). We did not observed a change in vascular permeability in nicotine pre-exposed sham animals compared to saline infused sham group as measured with vascular space marker ^14^[C]-sucrose in the perfusion medium. However, we did observe increased vascular permeability with or without nicotine pre-exposed tMCAO animals compared to saline-treated sham animals (*P* < 0.05) (Figure 2B).

**Table 1:** **Influx rate of D-glucose across blood–brain barrier in mice pre-infused with saline or nicotine and subjected to transient middle cerebral artery occlusion (tMCAO) or sham operation**

**Animal group**	**Saline infused**		**Nicotine (4.5 mg/kg/day)**	
	**Sham**	**tMCAO**	**Sham**	**tMCAO**
Rate of influx (*K*_*in*_) (mL.sec^−1^.g^−1^)	0.0026 ± 0.00069	0.0037 ± 0.00126^#^	0.0012 ± 0.00015**	0.0016 ± 0.00028***

Data represent mean ± SEM, n = 5–6 animals**,** **P < 0.01 and ***P < 0.001 comparing the nicotine-infused group and the corresponding saline-infused sham group, ^#^ *P* < 0.05 comparing the nicotine-infused tMCAO group and saline-infused sham group using one way ANOVA analysis of the variance and Bonferonni’s multiple comparison test.

#### Pre-exposure to nicotine for 14 days reduces ischemic-reperfusion-enhanced glucose transporter (GLUT1) expression

**Figure 3:**
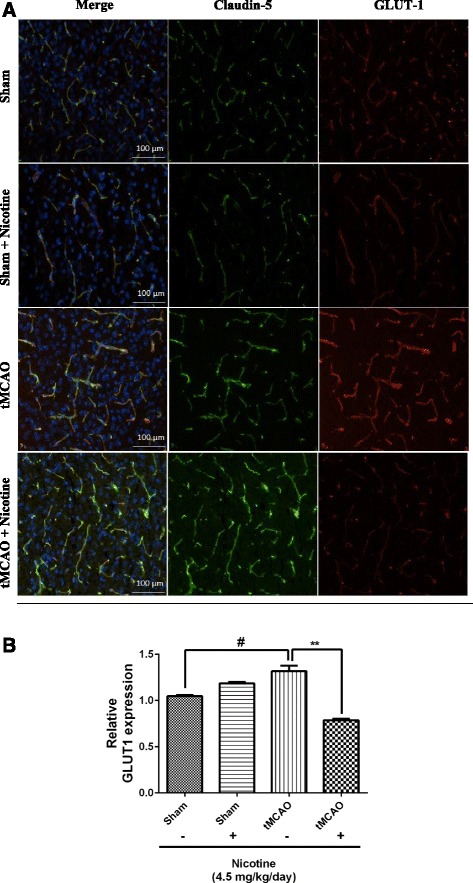
Pre-exposure to nicotine for 14 days reduces ischemic-reperfusion-enhanced glucose transporter (GLUT1) expression. **(A)** Effect of nicotine on brain microvascular GLUT1 expression in mouse brain. **(B)** Relative intensity of GLUT1 staining. Data represent mean ± SEM, n = 4–5, **P < 0.01 comparing the nicotine-infused group and the corresponding saline-infused sham group, ^#^ *P* < 0.05 comparing the nicotine- infused tMCAO group and saline-infused sham group using one way ANOVA analysis of the variance and Bonferonni’s multiple comparison test.

To test whether reduced BBB glucose transport activity with nicotine pre-exposure is an effect of changed GLUT1 expression in brain endothelial cells, we performed immunofluorescence analysis of cortical penumbral region of brain sections obtained from sham and tMCAO animals with or without pre-exposure to nicotine. To estimate the changes in brain vascular GLUT1 (red-color) expression, brain sections were also co-labeled with a brain vascular marker claudin-5 (green-color) (Figure 3A). Compared to saline-infused sham group animals, we observed a significant (*P* < 0.05) elevation in GLUT1 brain vascular expression in tMCAO animals and this was significantly (*P* < 0.01) down-regulated to normal levels in pre-exposed nicotine tMCAO animals (Figure 3B), consistent with that seen in the representative immunofluorescence brain images. These observations further suggest that the effect of nicotine pre-exposure on glucose transport is due to changes in GLUT1 protein levels in brain vasculature.

## Discussion

In our studies, we specifically determined unidirectional glucose transfer kinetics using an *in situ* brain perfusion method. In that method, we can precisely study and control substrate influx across the BBB since the perfusion medium is infused with a known concentration of glucose, at a controlled infusion rate, for a defined interval of time, without any systemic interference as seen with single intra-arterial injection in intact animals. Previously, other reports have shown that both acute and chronic administration of nicotine decreases glucose transport but increases glucose utilization in normal rat brain [22,23]. Similar to previously published glucose transporter kinetics, our data further reinforces that nicotine reduces glucose transfer rates across the BBB with no change or a slight increase in total vascular glucose transporter densities in nicotine-exposed sham animals. The rate of D-glucose influx in saline-infused sham animals is also in agreement with values for unidirectional blood–brain glucose transfer obtained in our laboratory [3] and by others in rats [23,32,33]. Our study further confirms enhanced GLUT1 transport influx rates and expression in ischemic mice compared to saline-infused sham animals [34]. Interestingly, in our studies, 14 day nicotine exposure before tMCAO reduced the ischemia-enhanced glucose transport rate across the BBB. Chronic nicotine exposure has been shown to affect multiple transporter systems at the BBB and in brain in physiological and pathophysiological conditions. Reduced expression of Na, K-ATPase at the BBB by chronic nicotine administration has been demonstrated to increase focal cerebral ischemic injury in rats [17] and a similar trend was observed in cultured brain endothelial cells exposed to hypoxia/aglycemia with or without nicotine and cotinine [14]. Nicotine has been shown to dose-dependently inhibit the increased NKCC activity observed during hypoxia/aglycemia in *in vitro* bovine brain microvascular cells [14]. Moreover, nicotine exposure increased both edema and infarct volume and worsened neurobehavioural outcomes in a 24 h permanent MCAO mouse model [16]. Importantly, the availability of glucose during and after stroke dramatically affects ischemic outcome [35-38]. Numerous studies examining different glucose-lowering strategies in patients with pre and post-ischemic hyperglycemia have been summarized in a recent review [39]. These studies indicate that tight glucose control is associated with a major risk of severe symptomatic and asymptomatic hypoglycemic episodes which further worsens the stroke injury [40-45]. Perhaps, the extension of observed reduced glucose transport in nicotine pre-exposed animals, across the BBB under normal conditions and during ischemic reperfusion may create a more glucose deprived state at the NVU and an exaggeration of ischemia-induced brain injury.

In this study, the direct mechanisms underlying nicotine-induced changes in GLUT1 transporter function or expression were not determined and they will be the focus of future studies. However, several mechanistic investigations from our lab have shown that nicotine can inhibit hypoxia-induced increased NKCC activity via a PKC mediated phosphorylation pathway [46]. We have also demonstrated an inhibitory effect of nicotine on several PKC isoforms during hypoxic conditions in bovine brain microvascular endothelial cells [46]. Similarly, other *in vitro* studies have indicated PKC-mediated glucose transporter activity in a variety of different tissues [47] and retinal capillaries [48]. Specifically in endothelial cells, PKC mediated increased glucose transport via translocation modulation has been demonstrated [49,50]. Likewise, in human brain, PKC-mediated GLUT1 expression has been suggested based on simultaneous changes in PKC co-localization with changes in GLUT1 density within endothelial domains [51]. Thus, we speculate nicotine-induced PKC changes could alter GLUT1 translocation in endothelial cells and may explain the possible reduced glucose transfer rate in the presence of no overall change or a slight increase in glucose transporter densities in brain capillary endothelial cells as observed in our nicotine-infused sham animals.

It is important to note that nicotine administered at 4.5 mg/kg/day for 1 day, 1 week, and 2 weeks through Alzet minipumps, results in plasma levels of nicotine of 80–100 ng/mL and the major metabolite cotinine of >250 ng/mL, similar to a heavy smoker [16]. A study by Abbruscato, et al., (2002) has previously demonstrated the direct dose-dependent effects of nicotine and cotinine on increased paracellular BBB permeability and reduced tight junctional protein expression, using bovine brain microvascular endothelial cells in culture, an *in vitro* BBB model. [13]. Also, others have demonstrated that plasma levels of nicotine and cotinine equivalent to smokers causes an increase in paracellular permeability in rats when exposed acutely (12 hours) [52]. In contrast, other studies have shown that a chronic nicotine dose (28 days) of 4.5 mg/kg/day does not affect the BBB integrity measured by sucrose [53]. Similar to previous reports, our results also suggest no change in the BBB integrity in nicotine pre-exposed animals. We calculated a glucose transport rate (*K*_*in*_) that was corrected for vascular space measured simultaneously in the same animals. We confirmed that the observed reduced glucose transport rate in ischemic animals was due to down-regulation of GLUT1 proteins in brain endothelial cells and not to changes in vascular volume, further validating the observed effect of nicotine on glucose transporter densities in brain [22]. Further, 4.5 mg/kg/day of nicotine does not affect cerebral blood flow as measured with *in situ* brain perfusion using radiotracer diazepam [54]. Moreover, we believe that glucose influx *K*_*in*_ is independent of blood flow since estimated initial influx rate is relatively small compared to estimated cerebral blood flow in the mouse under similar conditions.

## Conclusions

In conclusion, our observations validate an additional mechanism of brain endothelial cell GLUT1 modulation by nicotine exposure in brain during stroke. Given the past and present clinical, and preclinical observations of increase global cerebral glucose utilization and reduced glucose transport in nicotine-exposed subjects, together with our results warrants retrospective investigation of clinical studies examining glucose regulation in acute stroke patients with respect to tobacco use and careful design of possible future trials aiming to control glucose levels in the subset of ischemic and post-ischemic patients that are smokers.

## Abbreviations

BBB: Blood–brain barrier
CCA: Common carotid artery
ECA: External carotid artery
GLUT1: Glucose transporter-1
ICA: Internal carotid artery
IR: Ischemia/reperfusion
LCBF: Local cerebral blood flow
MCA: Middle cerebral artery
NVU: Neurovascular unit
tMCAO: Transient middle cerebral artery occlusion
